# P-97. Early Oral versus Prolonged Intravenous Antibiotics for Osteomyelitis (OVIVA-VA): A Single-Center Veterans Affairs Cohort

**DOI:** 10.1093/ofid/ofaf695.326

**Published:** 2026-01-11

**Authors:** Rishi Chanderraj, Elizabeth A Scruggs-Wodkowski, Kathleen A Linder, Louis Saravolatz, Stephen M Maurer, Nate Soper, Sandro Cinti, Emily Abdoler, Andrea Starnes, Jacob John, Kimberly Nofz, Sharon Thomas, Robert Woods, Ronald E Kendall

**Affiliations:** University of Michigan, Ann Arbor, MI; Veteran Affairs Ann Arbor Healthcare System; University of Michigan Medical School, Ann Arbor, Michigan; University of Michigan/Ann Arbor VAMC, Ann Arbor, Michigan; Michigan Medicine, Ann Arbor, Michigan; University of Pittsburgh Medical Center, South Burlington, Vermont; Trinity Health System, Ann Arbor, Michigan; University of Michigan, Ann Arbor, MI; University of Michigan, Ann Arbor, MI; Ann Arbor Veteran's Affairs Hospital, Ann Arbor, Michigan; Ann Arbor Veteran's Affairs Hospital, Ann Arbor, Michigan; Veterans Affairs VA Ann Arbor Healthcare System, Ann Arbor, Michigan; Veterans Affairs VA Ann Arbor Healthcare System, Ann Arbor, Michigan; University of Michigan, Ann Arbor, MI; VA Ann Arbor Healthcare System, Ann Arbor, Michigan

## Abstract

**Background:**

The VA manages high burdens of chronic osteomyelitis (OM). The OVIVA trial demonstrated that an early switch to oral antibiotics is not inferior to extended intravenous (IV) therapy in a predominantly civilian population. Whether a similar benefit holds within VHA remains uncertain.

**Figure:**
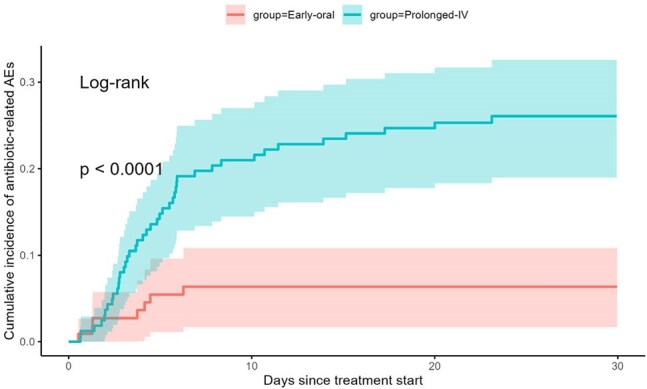
Early Oral Therapy Associated With Fewer Antibiotic-Related Adverse Events Thirty-day cumulative incidence curves demonstrate markedly fewer antibiotic-related adverse events in the early-oral group (5.3%) than in the prolonged-IV group (24.8%). Shaded ribbons depict 95 % confidence intervals; log-rank test p < 0.001.

**Figure:**
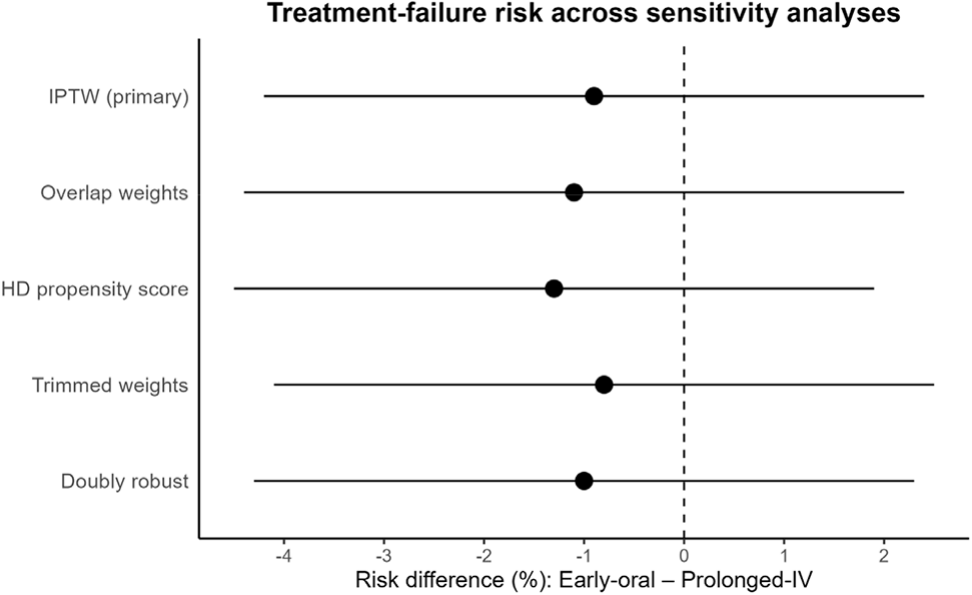
Non-Inferiority Stands Up to Every Sensitivity Check Forest plot of risk-difference estimates (early oral – prolonged IV) for treatment failure across five weighting/adjustment strategies. Point estimates remain close to zero, and all 95 % confidence intervals sit well within the ±7.5-percentage-point non-inferiority margin, confirming the robustness of the primary finding.

**Methods:**

We performed a retrospective single-center cohort study of Veterans with confirmed OM from 1/2017 – 12/2021 at the Ann Arbor VA. We compared patients treated with ≥4 weeks of IV antibiotics to those switched to an oral regimen within 7 days with inverse-probability-of-treatment weighting (IPTW). The primary endpoint was treatment failure within 1 year. Secondary endpoints were antibiotic-related adverse events, catheter-related complications, *C. difficile* infection, and hospital length of stay.

Baseline Characteristics and Covariate Balance After IPTW

**Figure:**
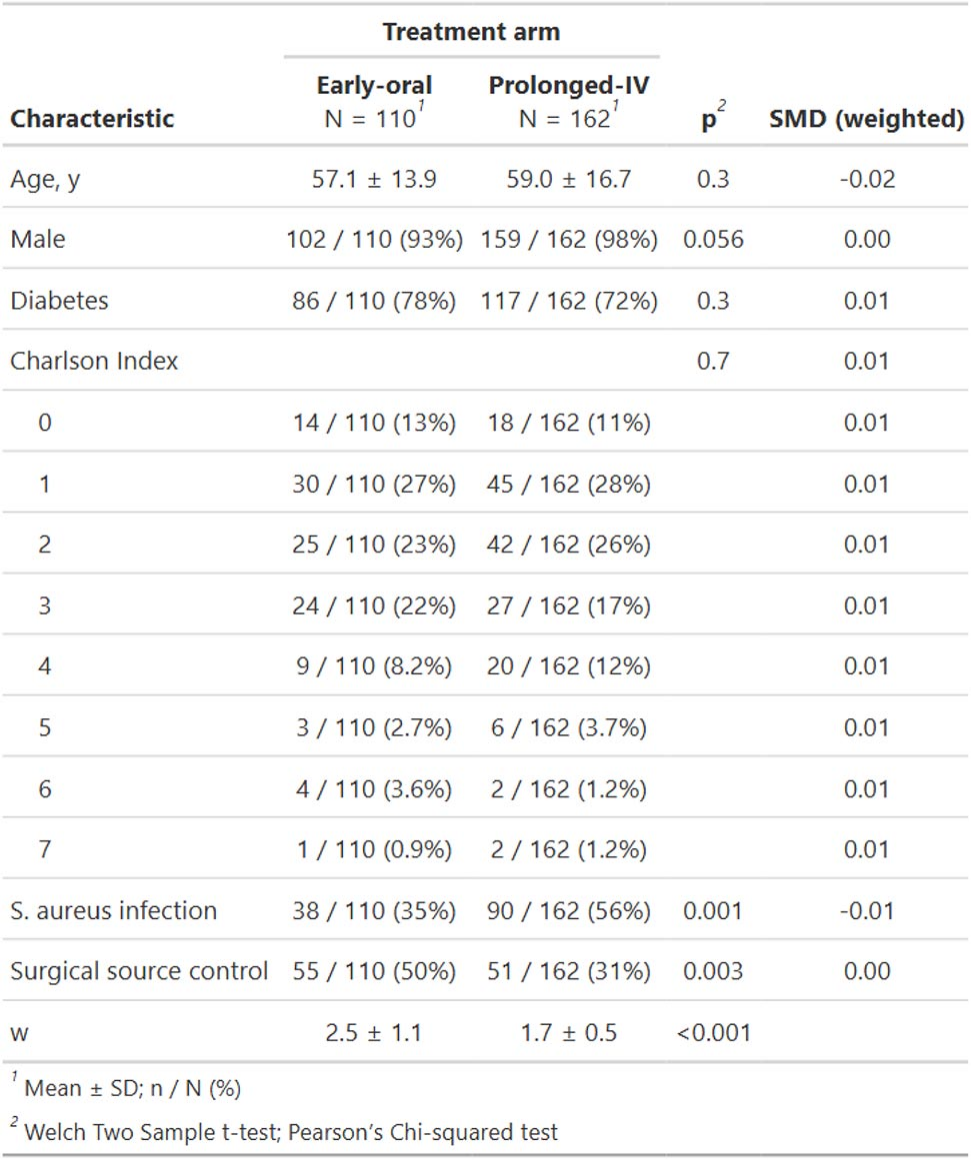

Unweighted patient demographics, comorbidities, and infection features (mean ± SD or n/N %) are compared between early-oral and prolonged-IV groups, with χ² or t-test p-values. The final column shows weighted standardized mean differences (SMDs) demonstrating excellent covariate balance (all SMDs ≤ 0.08) after inverse-probability treatment weighting.

**Results:**

We identified 110 patients treated with early oral antibiotics and 162 patients treated with prolonged IV antibiotics. There were higher rates of *S. aureus* infection lower rates of surgical source control among patients treated with early extended IV antibiotics. Imbalances were eliminated after weighting (weighted SMDs ≤ 0.08 for all covariates). After weighting, treatment failure occurred in 24/110 (21.8 %) patients on early-oral vs 41/162 (25.3 %) patients on prolonged-IV treatment (risk difference –0.9 pp, 95 % CI –4.2 to 2.4). Antibiotic-related adverse events were 5.3 % in the early-oral group vs 24.8 % in the prolonged IV group (–19.5%, 95 % CI –25.2 to –13.8); C. difficile 0 % vs 6.2 % (–6.2%, 95 % CI –8.6 to –3.8). Adjusted length of stay was 7.4 in the early oral group vs 12.2 days in the prolonged IV group (difference –4.8, 95 % CI –6.1 to –3.4). Sensitivity analyses using overlap weights, high-dimensional propensity scores, and weight trimming produced concordant estimates, and doubly robust models confirmed the findings.

**Conclusion:**

In this single-center VHA cohort, an early switch to oral antibiotics was associated with infection outcomes that were non-inferior to prolonged IV therapy and clinically meaningful reductions in antibiotic-related adverse events, catheter-related complications, *C. difficile infections*, and hospital length of stay. The benefits reported in OVIVA likely translate to routine practice at the VHA, supporting the adoption of an early oral step-down strategy.

**Disclosures:**

All Authors: No reported disclosures

